# No evidence for an other-race effect in dominance and trustworthy judgements from faces

**DOI:** 10.1177/03010066241258204

**Published:** 2024-06-17

**Authors:** Ao Wang, Bartholomew P.A. Quinn, Hannah Gofton, Timothy J. Andrews

**Affiliations:** University of York, UK

**Keywords:** faces, ORE, face impression, cross-cultural

## Abstract

A variety of evidence shows that social categorization of people based on their race can lead to stereotypical judgements and prejudicial behaviour. Here, we explore the extent to which trait judgements of faces are influenced by race. To address this issue, we measured the reliability of first impressions for own-race and other-race faces in Asian and White participants. Participants viewed pairs of faces and were asked to indicate which of the two faces was more dominant or which of the two faces was more trustworthy. We measured the consistency (or reliability) of these judgements across participants for own-race and other-races faces. We found that judgements of dominance or trustworthiness showed similar levels of reliability for own-race and other-race faces. Moreover, an item analysis showed that the judgements on individual trials were very similar across participants from different races. Next, participants made overall ratings of dominance and trustworthiness from own-race and other-race faces. Again, we found that there was no evidence for an ORE. Together, these results provide a new approach to measuring trait judgements of faces and show that in these conditions there is no ORE for the perception of dominance and trustworthiness.

When we encounter unfamiliar people, one of the most salient sources of information about that person is their face. From their face, we can form an impression of their gender, age and ethnicity (Bruce & Young, 2012). We can also make more subjective judgements about the character of a person (Todorov et al., 2015; Zebrowitz, 2017). Despite limited evidence about the accuracy of these first impressions, they are reliable across observers and can have important consequences in the real world (Olivola & Todorov, 2010; Rule & Ambady 2011; Zebrowitz & McDonald, 1991). For example, impressions of competence from facial photographs of politicians have been shown to predict the outcome of elections (Olivola et al., 2014). Recent behavioural models of facial impressions suggest that these judgements are based on three key dimensions: trustworthiness, dominance and attractiveness (Sutherland et al., 2013; Todorov et al., 2015). Dominance and trustworthiness are linked to the evaluation of competence and threat (Fiske et al., 2007), while attractiveness is linked to reward (Rhodes, 2006; Sutherland et al., 2013). As these dimensions can explain a large proportion of the variance across different trait judgements, they have formed an influential theoretical framework in face perception (Todorov et al., 2015).

A potential limitation in our understanding of facial impressions is that the majority of studies involve judgements of White faces with White participants (Jenkins et al., 2011). So, it is not clear whether similar trait judgements are evident for faces from other races or when faces are viewed by participants of a different race. It is well-established that the perception of own-race faces is better than for other-race faces (Malpass & Kravitz, 1969; Meissner & Bringham, 2001). Although the other-race effect (ORE) has mostly investigated the perception of identity, other studies have shown an ORE for the perception of facial expression (Jack & Schyns, 2017; Jack et al., 2012; Yan et al., 2016a, 2016b; Yuki et al., 2007). The facial cues that are used for trait judgements have been shown to be dependent on invariant aspects of faces such as gender and age, as well as changeable aspects of faces, such as expression (Oosterhof & Todorov, 2008; Sutherland et al., 2013; Vernon et al., 2014). This suggests that first impressions may differ for own-race faces compared to other-races faces.

The effect of race on the perception of facial impressions may also be affected by social categorization and in-group bias (Cook & Over, 2021). For example, individuals attribute positive characteristics to members of their own group, but have a less favourable perception of individuals who are not in their group (Allport, 1954; Sherif et al., 1961; Tajfel & Turner, 2004). As social interactions often begin with the face, the categorization of other-race faces as part of the outgroup may lead to negative stereotypes that could have an effect on trait judgements (Amodio, 2014; Fiske et al., 2007). Indeed, it has been argued that the facial properties, such as skin colour, that are important for social categorization may dominate other sources of facial information when trait judgements are made across individuals in the wider population (Cook & Over, 2021).

A number of studies have begun to investigate the effect of race in trait judgements of faces (Charbonneau et al., 2020; Jones et al., 2021; Short et al., 2012; Stanley et al., 2011; Sutherland et al., 2018; Xie et al. 2019; Zebrowitz et al., 1993, 2010). These studies have explored the extent to which dimensional models of first impressions might vary across different races. They show some cross-cultural similarities as well as some cross-cultural differences in the trait judgements (Jones et al., 2021; Sutherland et al., 2018). However, given the variation across faces within a race, it is possible that the level of cross-cultural differences may also be influenced by variance in the images used in different image sets (Xie et al., 2019). For example, it is known that ratings of attractiveness vary dramatically across faces from the same race and indeed from different images of the same person (Jenkins et al., 2011).

The aim of this study was to directly compare the trait judgements of own-race and other-race faces. In the first experiment, participants compared pairs of faces and had to decide which face was more dominant or trustworthy than the other. Reliability was determined for each face pair by measuring how often participants chose the same face as being more dominant or trustworthy. We then asked whether reliability for own-race faces was greater than for other-race faces. In the second experiment, we asked whether there were differences in the rating of own-race and other-races faces. A cross-over design was used to measure the performance of East-Asian and White participants when they viewed East-Asian, Black and White faces. These stimuli used in this study have previously been shown to demonstrate an ORE with behavioural task of face identity (Wang et al., 2022) and in the spatial pattern of response in the face regions of the human brain (Wang et al., 2023). Given the established ORE for these stimuli, our prediction was that participants should have lower reliability and lower overall rating for trait judgements of other-race faces. However, it is possible that an ORE could be evident in one task but not the other. For example, there could be no difference in the overall rating of traits in own-race and other-race faces, but the inter-rater reliability could be lower for own-race faces. This would suggest that there are differences in the variability in which faces are encoded across participants for own-race and other-race faces.

## Methods

### Participants

In Experiment 1, we recruited an opportunity sample of 128 participants (68 Asian: 43 females, mean age: 23.8; 60 White: 51 females, mean age: 18.9) for this study. Sixty-two participants (32 Asian, 30 White) were assigned to the dominance group and 66 participants (36 Asian, 30 White) were assigned to the trustworthy group. All Asian and White participants had grown up in East Asian and Western European countries, respectively. For Asian participants, their average time in the UK period was about 12 months (Mean ± SEM: 12.6 ± 1.36). In Experiment 2, we recruited an opportunity sample of 40 participants (20 Asian: 16 females, mean age: 20.0, 20 White; 16 females, mean age: 19.3) all of whom made both dominance and trustworthy judgements. All Asian and White participants had grown up in East Asian and Western European countries, respectively. For Asian participants, their average time in the UK period was about 13 months (Mean ± SEM: 13.2 ± 1.66). All participants gave their written informed consent. The study was approved by the Psychology Ethics Committee at the University of York. Participants were compensated with course credit or a voucher for participation.

### Stimuli

Images of White faces were taken from the Models Face Matching Test (Dowsett & Burton, 2015). Images of Asian and Black faces were taken from a variety of sources on the internet (Wang et al., 2022). A computational analysis of image properties using a deep convolutional neural network shows that the faces used in this study can be discriminated by their race (Wang et al., 2023). The images were cropped to 158 × 222 pixels. At a viewing distance of approximately 57 cm, each image subtended 7.8 × 10.2 degrees of visual angle. Experiments were performed online using Pavlovia.

### Experiment 1

In Experiment 1, we measured the reliability of trait judgements to own-race and other-race faces. Participants were assigned to a dominance or trustworthy group. There were 180 images for each task, which were arranged in 90 face pairs. Half of the trials were faces with the same identity and half of the trials were faces from different identities. The face pairs used in different trials were identical to those used in previous study that showed an ORE for matching identity (Wang et al., 2022). The same face pairs were used for dominance and trustworthiness judgements. On each trial, participants were asked to determine which face was more dominant or more trustworthy. The tasks were self-paced and new trials would only appear after a response had been made. Participants performed judgments on Asian, Black and White faces in separate tasks. The order of tests was counterbalanced across participants.

We measured reliability of each item in the task. This was done by first calculating for each trial the proportion of participants that chose one or other image as being more trustworthy or dominant. The reliability measure was calculated by taking the absolute difference between this proportion and 0.5 (chance). This value was then multiplied by 2 to give reliability scores from 0–1.0. For example, if, for a given face pair, 50% of participants had chosen one face and 50% had chosen the other, there would be no consistency or reliability across participants, the reliability would be (0.5–0.5 * 2) 0. This was taken as baseline or chance performance. If on another trial one face was perceived by participants to be more dominant on 100% of trials, the reliability would be (1.0–0.5 * 2) 1.0. This allowed us to calculate the average reliability across trials for either dominance or trustworthy judgements for the Asian or White participants for each of three tasks. It also allowed us to correlate reliability values across participant groups and judgements of trustworthiness or dominance with the same images.

### Experiment 2

In Experiment 2, we measured the absolute level of dominance or trustworthiness to the same own-race and other-race faces used in Experiment 1. Participants viewed individual faces in two separate blocks. Each block contained 90 Asian, 90 Black and 90 White faces. Different face images were used in each block. In one block, they were asked to rate the level of dominance and in the other block they were asked to judge the trustworthiness. The tasks were self-paced and new trials would only appear after a response had been made. Participants made judgements on a 9-point Likert scale. The order of blocks and collection of 270 faces appearing within each block were evenly counterbalanced across participants.

## Results

### Experiment 1

**Figure 1.**
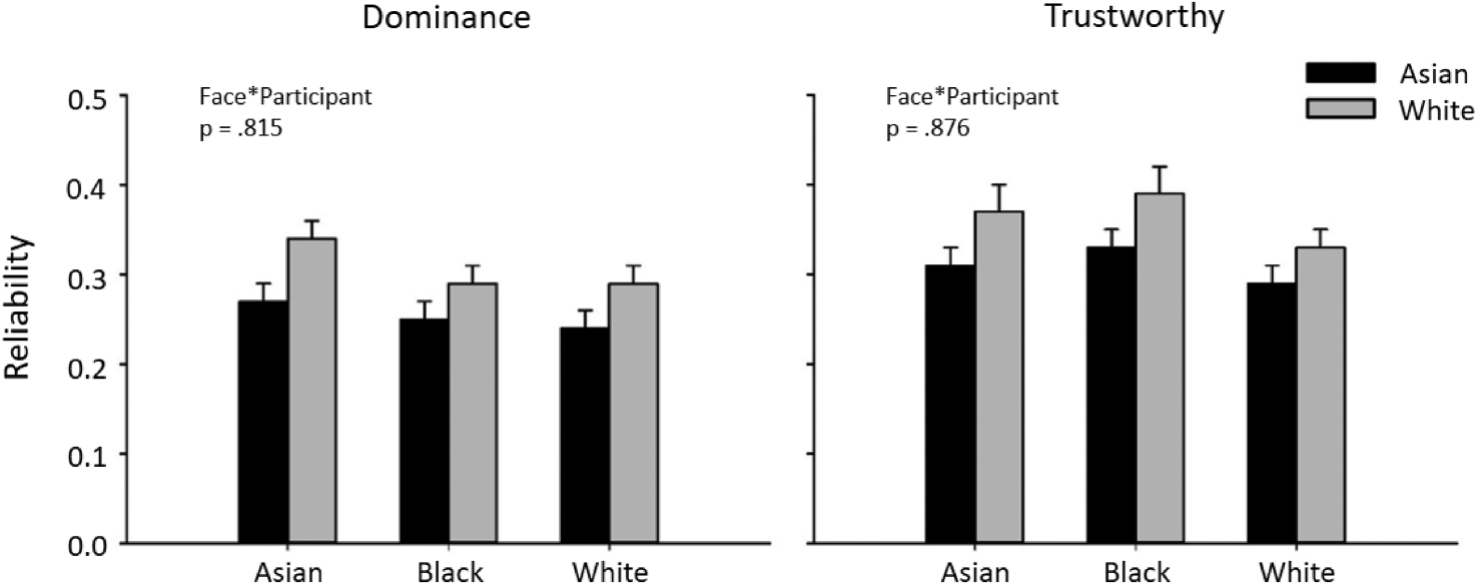
Reliability on the dominance and trustworthy task with Asian and White participants viewing Asian, Black and White faces. Chance level is 0. The data show no significant effect of the participant race. Error bars show +1 SEM.

In this experiment, we asked whether there were differences in the reliability of first impressions to own-race and other-race faces. We calculated the average reliability for judgements of trustworthiness or dominance in each participant group (Asian, White) for each test (Asian, Black, White). Figure 1 shows the average reliability of Asian and White participants in the dominance and trustworthy tasks. It is clear from the graphs that average reliability scores were significantly greater than chance (Table 1). This shows that participants were consistent in their dominance and trustworthiness judgements. We then asked whether there was an ORE for the reliability of judgements of trustworthiness or dominance. 3 (Face: Asian, Black, White) × 2 (Participant: Asian, White) mixed effects ANOVA were then performed separately for the trustworthy and dominance groups to determine the effect of face race and participant race on trait judgements.

**Table 1.** T-test results of calculated reliability versus chance (0) for Asian and White participants viewing Asian, Black and White faces.

		Asian face		Black face		White face	
		t	*p*	t	*p*	t	*p*
**Asian**	Dominance	15.4	<.001	13.3	<.001	14.2	<.001
	Trustworthy	13.1	<.001	15.3	<.001	13.8	<.001
**White**	Dominance	15.5	<.001	14.5	<.001	14.9	<.001
	Trustworthy	14.5	<.001	15.1	<.001	14.2	<.001

For dominance judgements, there was a significant effect of Participant (F(1, 178) = 12.497, *p* < .001, Partial Eta Squared = .066), but no effect of Face (F(2, 356) = 3.036, *p* = .052, Partial Eta Squared = .017). The effect of participant was due to higher reliability scores for White participants (Mean ± SEM: 0.311 ± 0.021) compared to Asian participants (Mean ± SEM: 0.254 ± 0.018). However, there was no interaction between Face and Participant (F(2, 356) = .193, *p* = .815, Partial Eta Squared = .001). This shows that reliability judgements of dominance were not affected by participant race and that there is no evidence for an ORE for judgements of dominance.

For the trustworthy judgements, there was a significant effect of Participant (F(1, 178) = 9.491, *p* < .01, Partial Eta Squared = .051), but no effect of Face (F(2, 356) = 1.577, *p* = .208, Partial Eta Squared = .009). The effect of participant was due to higher reliability scores for White participants (Mean ± SEM: 0.365 ± .025) compared to Asian participants (Mean ± SEM: 0.308 ± 0.22). However, again there was no interaction between Face and Participant (F(2, 356) = .133, *p* = .876, Partial Eta Squared = .001). This shows that reliability judgements of trustworthiness were also not affected by participant race and there is no evidence for an ORE for trustworthy judgements.

**Figure 2.**
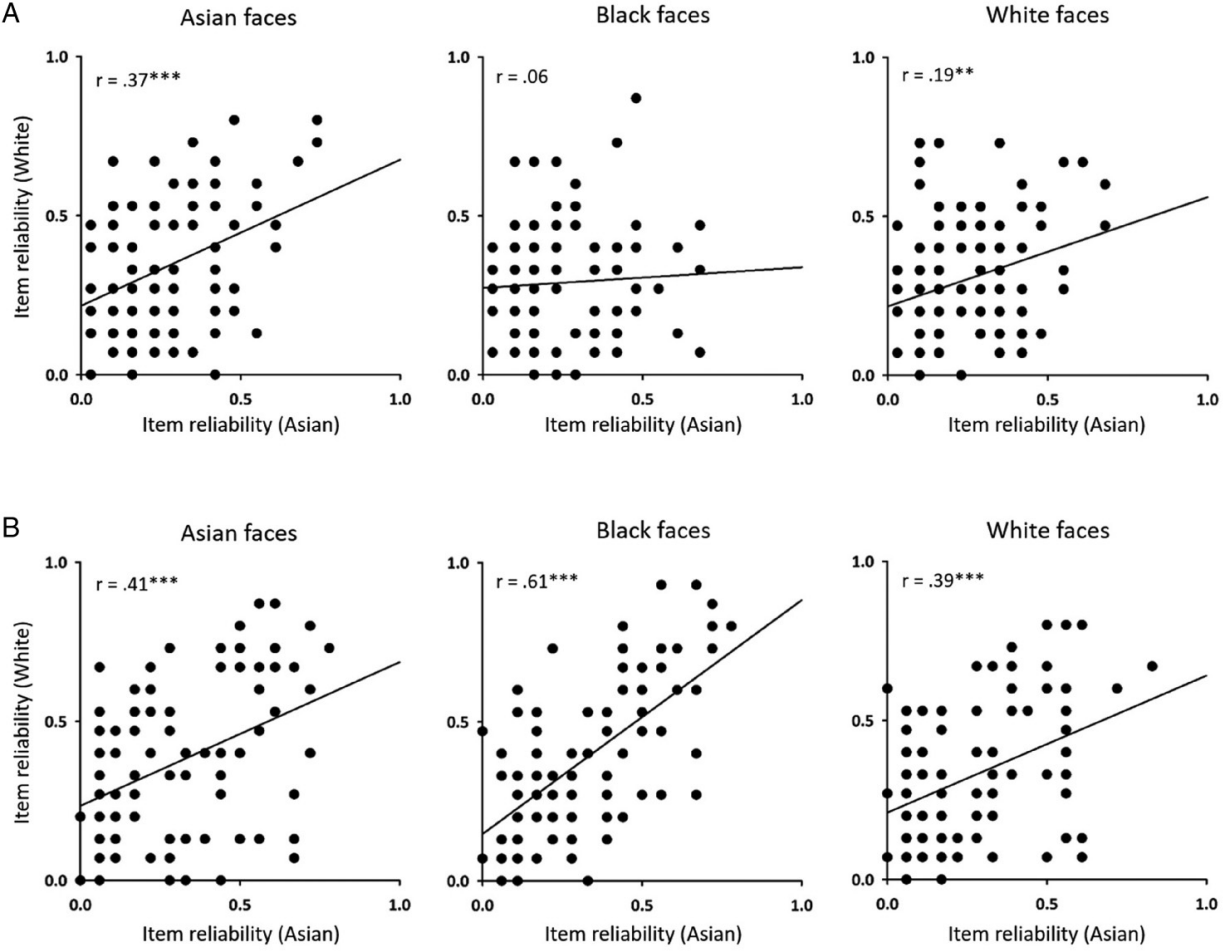
Correlation between item reliability of Asian, Black and White faces from Asian and White participants for judgments of (A) dominance and (B) trustworthiness. Significant positive correlations were found for each task for both own-race and other-race faces except black faces in dominance, suggesting a similar pattern of face impression formation for Asian and White participants. ** *p* < .01, *** *p* < .001.

Next, we asked if judgements of dominance or trustworthiness were similar across participants from different races. To do this, we correlated the reliability values from different race participants (Figure 2). For Asian and White faces, there were significant correlations between Asian and White participants for reliability scores on dominance (Asian: *r_s _*= .37, *p* < .001; White: *r_s _*= .19, *p* < .01) and trustworthy judgements (Asian: *r_s _*= .41, *p* < .001; White: *r_s _*= .39, *p* < .001). For Black faces, there was a significant correlation between White and Asian participants for trustworthy judgements (*r_s _*= .61, *p* < .001), but not for dominance judgements (*r_s _*= .06, *p* = .095). Overall these data show similar patterns of dominance and trustworthy judgements across Asian and White participants.

**Figure 3.**
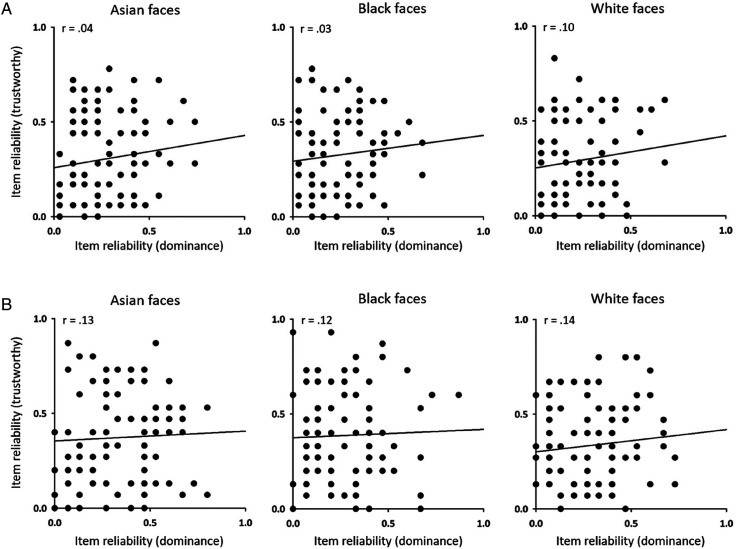
Correlation between the reliability of dominance and trustworthiness judgements for Asian, Black and White faces in (A) Asian and (B) White participants. No significant correlations were found.

We then asked whether reliability judgements of dominance and trustworthiness were linked (Figure 3). To do this, we correlated the reliability of dominance judgements with the reliability of trustworthy judgements across the same items for Asian (Figure 3A) or White (Figure 3B) participants. We found no correlation between the reliability of trustworthy and dominance judgements, which is consistent with the idea that these judgements are independent.

Finally, we investigated whether the identity of face pairs had any effect on the reliability of judgements of dominance and trustworthiness. Specifically, we asked if judgements on different identity trials were more reliable than for same identity trials. A 3 way (Face: Asian, Black, White) × 2 (Participant: Asian, White) × 2 (Identity: Same, Different) ANOVA was performed for Asian and White participants. We found no effect of Identity (F(1, 176) = .648, *p* = .691, Partial Eta Squared = .002) across all the combination of participant races and face races, which indicates the identity of face pairs does not influence the form of face impression for Asian and White participants.

### Experiment 2

**Figure 4.**
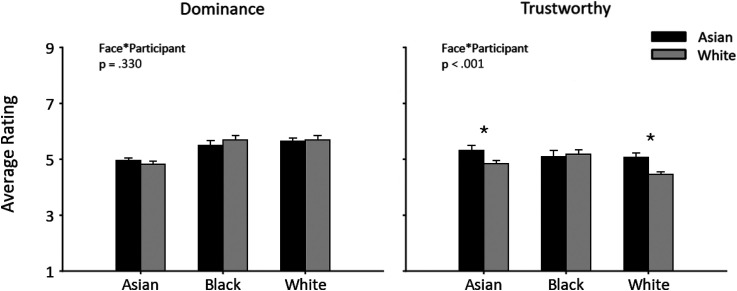
Average rating of dominance and trustworthiness with Asian and White participants viewing Asian, Black and White faces. Chance level is 0. The data show no significant effect of the participant race. Error bars show +1 SEM.

In this experiment, we asked whether there were differences in the overall rating of first impressions to own-race and other-race faces. We calculated the average rating of trustworthiness or dominance in each participant group (Asian, White) for each face race (Asian, Black, White). Figure 4 shows the average rating of Asian and White participants in the dominance and trustworthy tasks. We then asked whether there was an ORE for the reliability of judgements of trustworthiness or dominance. 3 (Face: Asian, Black, White) × 2 (Participant: Asian, White) mixed effects ANOVA were then performed separately for the trustworthy and dominance groups.

For dominance ratings, there was no effect of Participant (F(1, 38) = 0.05, *p* = .0.83, Partial Eta Squared = .00), but there was a significant effect of Face (F(2, 76) = 32.6, *p* < .001, Partial Eta Squared = .46). The effect of face was due to higher ratings for Black (Mean ± SEM: 5.60 ± 0.12) and White (Mean ± SEM: 5.68 ± 0.10) faces compared to Asian faces (Mean ± SEM: 4.89 ± 0.07). However, there was no interaction between Face and Participant (F(2, 76) = 1.12, *p* = .33, Partial Eta Squared = .03). This shows that the rating of dominance was not affected by participant race. Therefore, there is no evidence for an ORE for judgements of dominance.

For the trustworthy judgements, there was no effect of Participant (F(1, 38) = 2.79, *p* = .0.103, Partial Eta Squared = .07), but there was a significant effect of Face (F(2, 76) = 12.51, *p* < .001, Partial Eta Squared = .25). The effect of face was due to higher ratings for Asian (Mean ± SEM: 5.08 ± 0.11) and Black (Mean ± SEM: 5.14 ± 0.13) faces compared to White faces (Mean ± SEM: 4.76 ± 0.11). There was also an interaction between Face and Participant (F(2, 76) = 10.27, *p* < .001, Partial Eta Squared = .21). To explore this interaction, we compared ratings of trustworthiness for each face race. Asian participants rated both Asian and White faces as more trustworthy than White participants (Asian face: t(38) = 2.42 *p* = .021, *d* = 0.76; White face: t(38) = 3.06, *p* = .004, *d* = 0.97). However, the effect size was greater for White faces which is in the opposite direction for the ORE. There was no difference in the ratings of Black faces by Asian and White participants (t(38) = −0.32, *p* = .749, *d* = 0.1). These data, therefore, do not show any pattern that is consistent with a clear ORE for trustworthiness.

**Figure 5.**
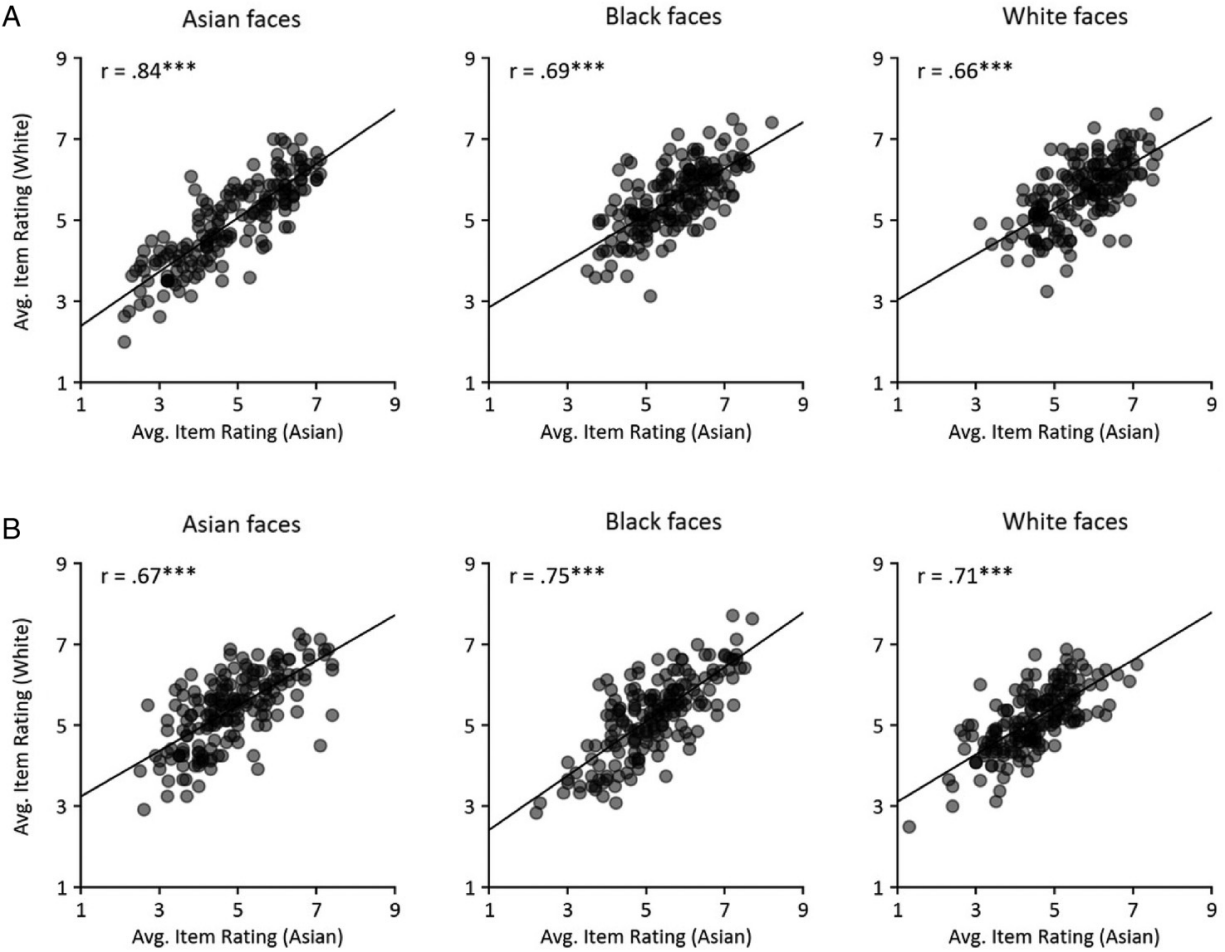
Correlation between average item rating of Asian, Black and White faces from Asian and White participants for judgements of (A) dominance and (B) trustworthiness. Significant positive correlations were found for each task for both own-race and other-race faces except black faces in dominance, suggesting a similar pattern of face impression formation for Asian and White participants.*** *p* < .001.

Next, we asked if judgements of dominance or trustworthiness were similar across participants from different races. To do this, we correlated the ratings of items from different race participants (Figure 5). For Asian, Black and White faces, there were significant correlations between Asian and White participants for ratings on dominance (Asian: *r *= .84, *p* < .001; Black: *r *= .69, *p* < .001; White: *r *= .66, *p* < .001) and trustworthy judgements (Asian: *r *= .67, *p* < .001; Black: *r *= .75, *p* < .001; White: *r *= .71, *p* < .001). Overall, these data show similar patterns of dominance and trustworthy judgements across Asian and White participants.

**Figure 6.**
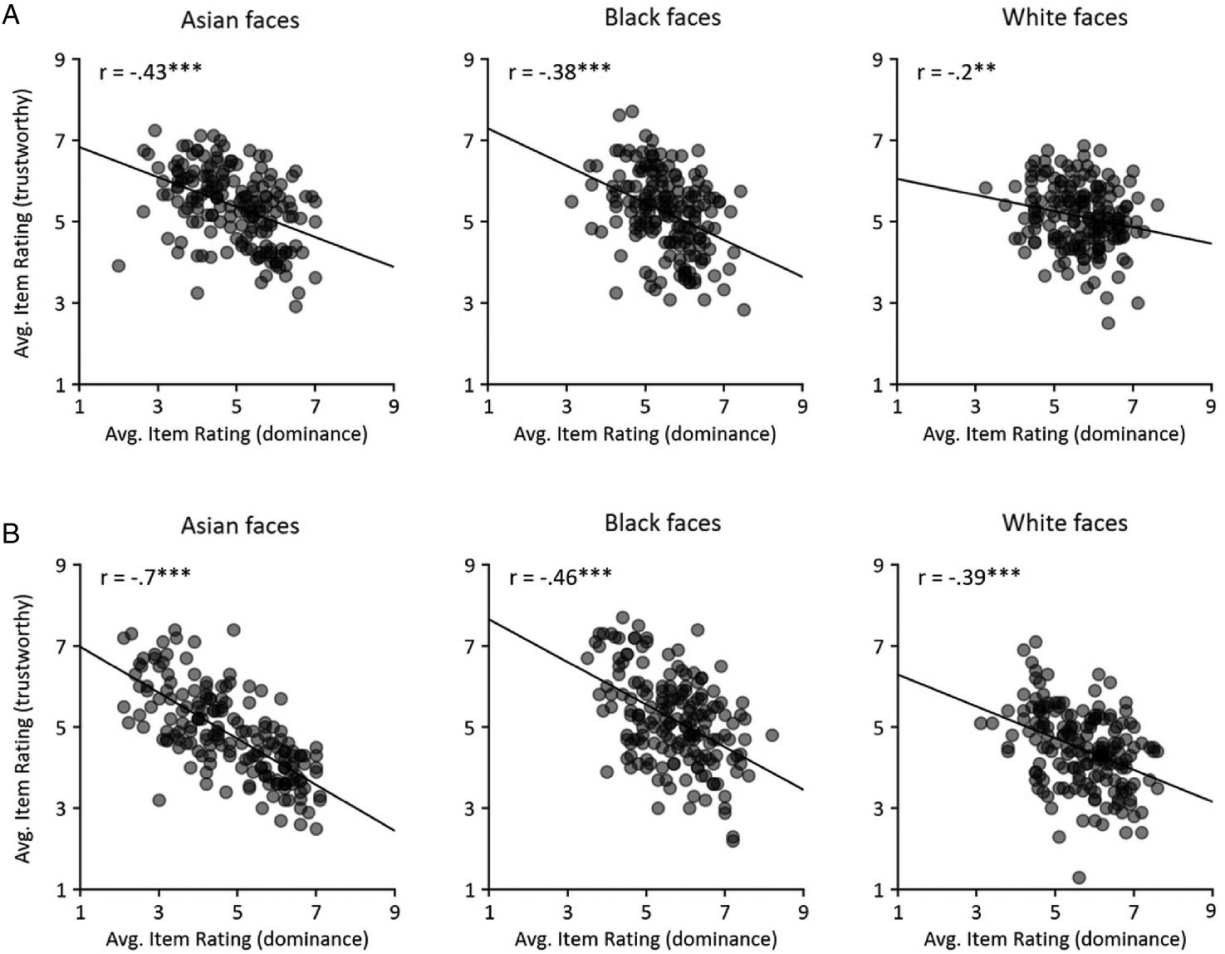
Correlation between the reliability of dominance and trustworthiness judgements for Asian, Black and White faces in (A) Asian and (B) White participants. No significant correlations were found. ** *p* < .01, *** *p* < .001.

We then asked whether the ratings of dominance and trustworthiness were linked (Figure 6). To do this, we correlated the ratings of dominance with the ratings of trustworthiness across items for Asian (Figure 3A) or White (Figure 3B) participants. We found a negative correlation between dominance and trustworthiness ratings for Asian faces (Asian participants: *r *= −.43, *p* < .001; White participants: *r *= −.70, *p* < .001), Black faces (Asian participants: *r *= −.38, *p* < .001; White participants: *r *= −.46, *p* < .001), White faces (Asian participants: *r *= −.20, *p* = .006; White participants: *r *= −.39, *p* < .001).

## Discussion

The aim of this study was to determine whether there was an ORE for trait judgements of faces. To address this question, we measured the reliability of judgements of dominance and trustworthiness judgments from East Asian and White participants while viewing East Asian, Black and White faces. Reliability was not significantly different between own-race and other-race faces. The reliability in the response to individual items was also similar across participants from different races. Consistent with reliability, we found no evidence that overall ratings of trustworthiness or dominance were higher for own-race faces compared to other-race faces. Taken together, we find no evidence for an ORE in trait judgements from first impressions of faces.

In Experiment 1, participants made relative judgements of either dominance or trustworthiness based on pairs of faces. A range of studies have shown that people are more accurate at perceiving the identity (Malpass & Kravitz, 1969; Meissner & Brigham, 2001) or facial expression (Elfenbein & Ambady, 2002; Jack et al., 2012; Yan et al., 2016a, 2016b) of own-race faces compared to other race faces. The faces used in this study were taken from matching tasks in which an ORE was previously found (Wang et al., 2022). In this study, participants had to decide whether the faces belonged to the same or a different identity. Here, participants had to decide which face was the most trustworthy or which face was the most dominant. We found that participants did not perform this task idiosyncratically, but showed a bias toward one face or the other. Nevertheless, we did not find that consistency or reliability of this bias was different for own-race faces or other-races faces.

There is mixed evidence for the role of culture or race in first impressions from faces. Some studies have found significant cross-cultural similarities when people make these trait judgements (Cunningham et al., 1995; Walker et al., 2011; Zebrowitz et al., 1993). For example, Zebrowitz and colleagues (1993) found high levels of intra-observer and inter-observer reliability in trait judgements of faces. However, other studies suggest that there are significant cross-cultural differences (Krys et al., 2014; Xie et al., 2019; Zebrowitz et al., 2012). For example, Xie and colleagues (2019) reported that the perceiver race and gender explained more of the variance than the face image when making trait judgements. A lack of convergence on the effect of race or culture on judgements of first impressions is also evident in data-driven models of first impressions. Some studies show evidence for common cross-cultural dimensions across a range of judgements (Sutherland et al., 2018), while other studies report regional differences (Jones et al., 2021).

One possible reason for variation across studies could be variance in the faces used in different image sets. In our study, participants from different races judged the same faces using a two-alternative forced choice. The advantage of this approach is that it provides an unbiased measure. Participants do not have to make absolute judgements with reference to an internal scale on the dimension that is being judged, but rather they just must make a relative judgement. Studies of sensory perception have shown that relative judgements are more accurate and reliable than absolute judgements (Andrews et al., 2001).

Nevertheless, it is possible that, despite similar reliability for own-race and other-race faces, there were differences in the overall rating of dominance and trustworthiness. In Experiment 2, we measured overall ratings of dominance and trustworthiness using the same images that were used in Experiment 1. We found that ratings of dominance and trustworthiness were highly correlated across individual face images. Moreover, we found no evidence that own-race faces were consistently rated more highly than other-race faces. For example, although Asian participants rated Asian faces to be more trustworthy than White participants, they also rated White faces as being more trustworthy. There were no differences in the ratings of dominance between Asian and White participants.

It is well-established that the categorization of people into social groups can lead to the development of inaccurate stereotypes, in which we perceive members of our own group more positively than members of other groups (Amodio, 2014; Fiske & Neuberg, 1990; Macrae & Bodenhausen, 2000). Individuals are often discriminated against because of their nationality, ethnicity, political ideology and sexual orientation (Cikara & van Bavel, 2014; Paluck, 2016; van Bavel et al., 2008). In many parts of Europe and in the USA, immigrants face rising hostility from the local population and support for explicitly racist political groups is increasing (Hainsworth, 2016). These biases emerge early in development and can be highly resistant to change (Bigler & Liben, 2007; Over & McCall, 2018). Our findings may provide a helpful outlook in attempts to reduce prejudice (Paluck, 2016) by showing that race-based stereotypes do not reflect cross-cultural differences at this level of perceptual processing.

In conclusion, our results show that using a novel 2AFC paradigm that there was no evidence that judgements of dominance and trustworthiness were more reliable for own-race faces. Similarly, there was no evidence that overall ratings were higher for own-race faces. Rather, the data showed that participants from different races perceived dominance and trustworthiness in own-race and other-race faces in a similar way.
